# Notes

**Published:** 1885-07

**Authors:** 


### EDITORIAL NOTES.

New York Polyclinic School of Medicine.—The pathological laboratory of this institution having been fitted up at great expense, we feel it a pleasure to be able to announce to our colleagues that the Faculty have consented to Dr. Billings forming special classes of Veterinarians, or taking private students in courses of Morbid Histology. Is is scarcely necessary to say that every endeavor will be made by Dr. Billings to further the studies and desires of Veterinarians in this matter. C.

American Veterinary College.—We are pleased to inform our readers that Dr. Billings has made such arrangement with Dr. Liautard that he will be enabled to give a continued series of lectures each week during the coming winter and spring courses in general pathology and demonstrative pathological anatomy. Autopsies at the school will be conducted by Dr. Billings upon every animal dying at the hospital. C.

